# The effect of ILs as co-salts in electrolytes for high voltage supercapacitors

**DOI:** 10.1038/s41598-018-37322-y

**Published:** 2019-02-04

**Authors:** Ha-Na Kwon, Su-Jin Jang, Yun Chan Kang, Kwang Chul Roh

**Affiliations:** 1 Energy Efficient Materials Team, Energy & Environmental Division, Korea Institute of Ceramic Engineering and Technology (KICET), Soho-Ro 101, Jinju-Si, Gyeongnam 52581 Korea; 2 Department of Materials Science & Engineering, Korea University, Anam-dong, Seongbuk-Gu, Seoul 02841 Republic of Korea

## Abstract

Ionic liquids (ILs) which have electrical stability are attractive materials to enhance the potential window of electrolyte. According to the potential window is extended, available voltage for supercapacitor is broaden. In this study, the addition of ILs which is 1-ethyl-3-methylimidazolium tetrafluoroborate (EMIBF4) and 1-ethyl-3-methylimidazolium bis(trifluoromethylesulfonyl) imide (EMITFSI) as co-salts, to a supercapacitor electrolyte increases the ionic conductivity and stability of it due to inhibition of electrolyte decomposition. As a result, the electrochemical stability potential windows (ESPWs) of supercapacitor is improved and the supercapacitor exhibited increased cycling stability. The loss of specific capacitance upon addition of 7 wt% EMIBF4 or EMITFSI to the electrolyte was 2.5% and 8.7%, respectively, after 10,000 cycles at 3.5 V, compared to the specific capacitance of the initial discharge.

## Introduction

Supercapacitors (SCs), also known as “electrochemical double-layer capacitors” (EDLCs), have attracted a great deal of interest in both academic and industrial research. This is mainly used due to the distinct advantages of SCs over batteries and fuel cells, including higher power densities that are induced by fast charging/discharging rates and longer cycle lives^1^. The energies and power densities of SCs depend on the squares of their applicable cell voltages. However, the electrochemical stability potential windows (ESPWs) of some SCs are limited by the decomposition of the electrolyte solvent^2^, and charging these devices beyond a specific voltage can cause the degradation of some components. Since electrolytes bear localised charges, they often have lower electrochemical stabilities than neutral solvent molecules; therefore, the choice of electrolyte can determine the ESPW of SCs^3^.

To overcome the drawbacks of organic solvents, ionic liquids (ILs), well-known room-temperature solvent-free electrolytes with wide ESPWs, have been used in SCs that operate at high voltages^4^. ILs exhibit many important properties, such as high electrochemical and thermal stabilities, while they are non-flammable and non-volatile. However, their ionic conductivities are only a few mS cm^−1^, since their high viscosities hamper the movements of ions in the electrolyte^5^.

Therefore, ILs have mainly been used at high temperatures rather than room temperature (RT). Ionic liquids (PP13TFSI, EMITFSI, etc.) in acetonitrile have previously been used as electrolytes in SCs with activated carbon at 60 °C^6^. Hybrid activated-carbon/conducting-polymer devices have exhibited improved performance at cell voltages greater than 3 V when used with 1-butyl-1-methylpyrrolidinium bis(trifluoromethylesulfonyl) imide (PYR14TFSI)^7,8^. The use of 1,2-dimethoxyethane (1,2-DME) in SCs has been shown to increase the ionic conductivity of 1-ethyl-3-methylimidazolium bis(trifluoromethylesulfonyl) imide (EMITFSI) by decreasing the viscosity of the IL^9^. Nevertheless, ILs still fail to meet the required performance criteria because of their generally higher operating temperatures (>RT) and the high amounts (>30 wt%) of ILs required in electrolytes, a consequence of low ionic conductivities and high viscosities. Using large amounts of ILs is burdensome because of their relatively high costs compared with conventional electrolytes such as 1 M TEABF4/PC^10,11^. In an effort to enhance this system, various studies into electrolyte performance aimed at improving the properties of SCs at high voltages have been conducted. Nevertheless, investigating suitable ILs for use in electrolytes and determining the amount of IL in an electrolyte that provides optimum performance at RT is also important. In this work, we employed ILs as electrolyte co-salts in order to broaden the ESPWs of SCs based on porous graphene. As a result, we determined the optimal proportions of ILs in SC electrolytes and identified the reasons for the observed increased stabilities at high voltages.

## Results and Discussion

**Figure 1:**
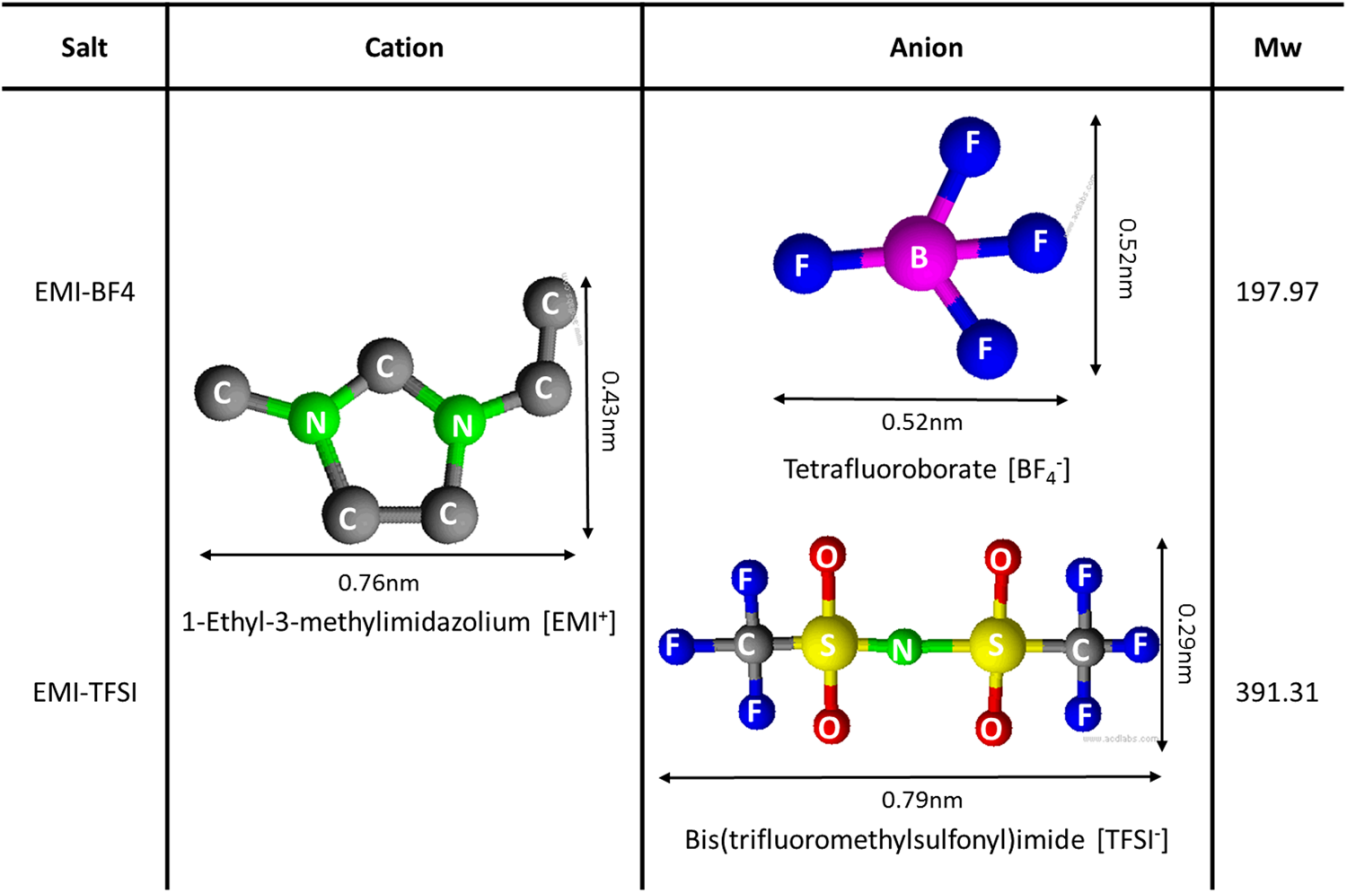
Molecular structures and weights of the cations and anions of the ILs employed in this study.

The ILs employed 1-ethyl-3-methylimidazolium tetrafluoroborate (EMIBF4, KOEI, Japan) and 1-ethyl-3-methyl-imidazolium bis(trifluoromethylesulfonyl)imide (EMITFSI, IoLiTec, Germany). Electrolytes were prepared by adding these ILs with organic electrolyte (1 M TEABF4/PC) at IL concentrations that ranged from 4 to 75 wt%. The cations and anions of ILs using co-salts in electrolyte are presented in Fig. 1^12,13^. Among ILs with superb electrochemical stabilities, EMI-based ILs were chosen because of their higher conductivities and lower melting points compared to other ILs, thereby making them suitable electrolytes for SCs that operate at high voltages. Porous graphene, which has a 2D structure and a large surface area, was employed as the electrode material since it facilitates the mobilities of ions in electrolytes that use ILs as co-salts^14^. The properties of porous graphene are studied and shown in Supplymentary (S1) and in order to remove residual moisture, the graphene was placed in a vacuum oven overnight at 120 °C, prior to use. All electrochemical experiments were performed using two- or three electrode systems at RT (Supplymentary, S2).

**Figure 2:**
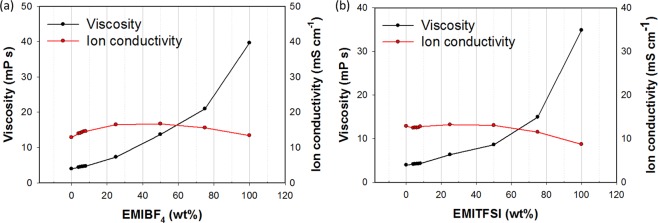
Relationships between IL wt%, viscosity and ionic conductivity: (**a**) EMIBF4 and (**b**) EMITFSI.

The concentrations and mobilities of ions are important factors that determine the ionic conductivities of electrolytes, since cations and anions are the charge carriers during SC operation^15^. The results reveal that the ionic conductivity of each electrolyte increased upon addition of the IL, to a maximum value at an IL content of 25 wt% (Fig. 2). This ascribable to the ILs creating additional cations and anions through the formation of ion pairs, which in turn increases the ion density of the electrolytes. More ions enter the variously sized pores of the porous graphene; consequently, a variety of ions are present in the electrolytes. This leads to improved SC capacitance due to the more-rapid ionic response compared to that of the neat organic electrolyte^16^.

On the other hand, the viscosities of the electrolytes were found to increase with increasing ILs content, which suppressed their ion mobilities (Fig. 2). The Nernst-Einstein equation reveals the correlation between the electro-conductivity of an electrolyte and viscosity, as well as the ionic radii, and molecular weights of the ions in the electrolytes^17,18^. The relationship between conductivity and viscosity is detailed in the Supplymentary (S3) based on the factors shown in Fig. 1. The electrode was difficult to impregnate with the electrolyte at high electrolyte viscosities, even though ions were highly concentrated due to the use of the ILs. This, in turn, increased the equivalent series resistance (ESR) and limited the rate and power performance^19,20^. In order to attain the required SC-electrolyte properties, 25 wt% is the preferred IL-concentration limit, based on the data in Fig. 2.

**Figure 3:**
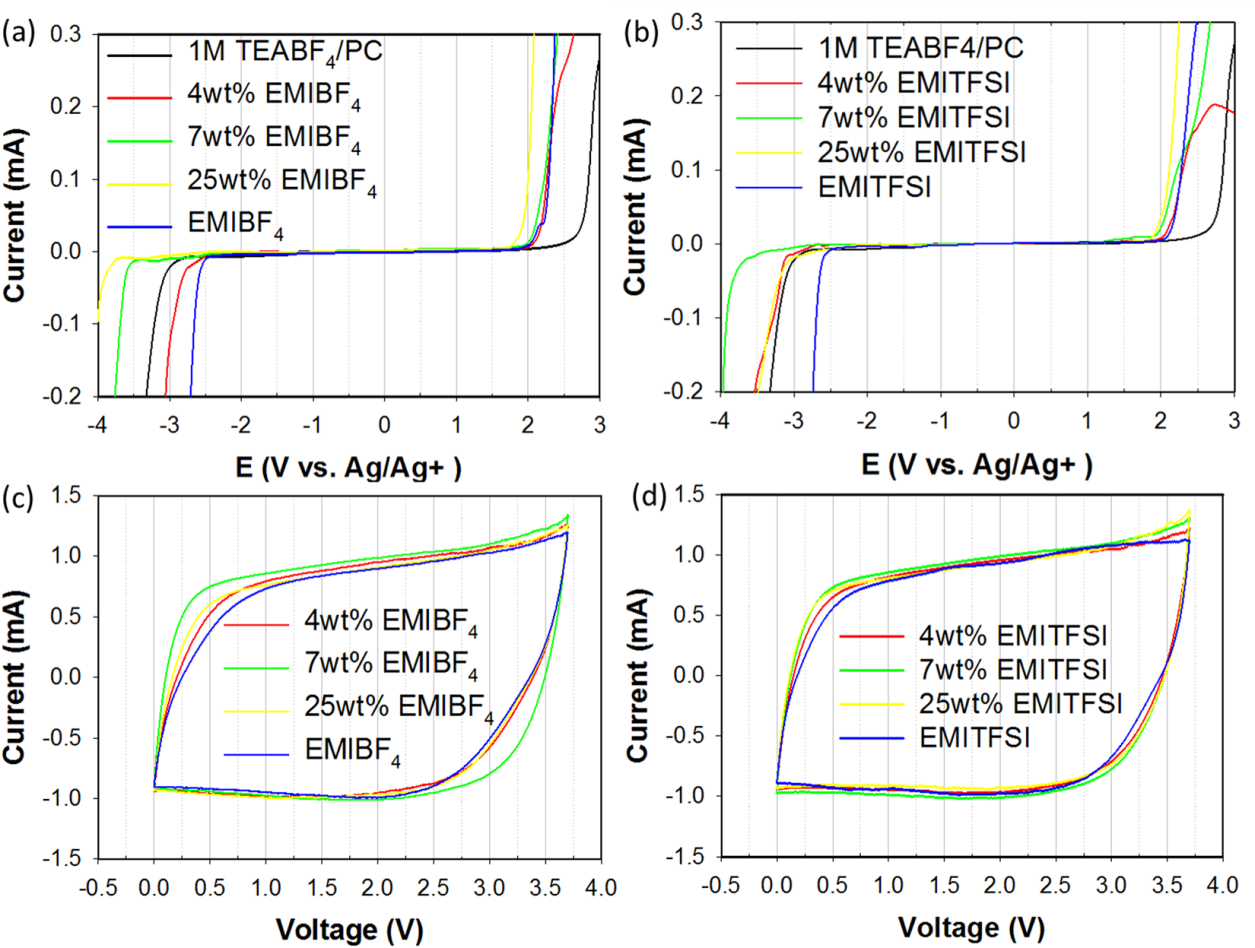
Linear-sweep voltammetry according to wt% of (**a**) EMIBF4 and (**b**) EMITFIS. Cyclic voltammetry of (**c**) EMIBF4 and (**d**) EMITFSI as co-salts at 3.7 V. Both sets of experiments were performed at a scan rate of 2 mV s^−1^.

Linear-sweep voltammetry (LSV) was used to determine the ESPW of the electrolytes and to explore the cathodic and anodic stability limits at a current density of 2 mA cm^−2^ (Fig. 3(a,b)). LSV is an effective analytical method that provides information about the oxidation (anodic limit) and reduction (cathodic limit) reactions of an electrolyte; as a consequence, the ESPW broadening of an electrolyte in response to the addition of an IL can be explored^21^. The electrolyte was cathodically stable to −3.5 V vs. Ag when 7 wt% EMIBF4 was added as a co-salt, while anodic decomposition was observed at above 2.0 V. In contrast, when 7 wt.% EMITFSI was added as a co-salt, the electrolyte decomposed at −3.7 V vs. Ag, while the electrolyte was anodically stable to 1.9 V vs. Ag. The ESPWs of these EMIBF4- and EMITFS-co-salt-containing electrolytes were 5.5 V and 5.6 V, respectively, which demonstrates that ILs promote wider ESPWs in electrolytes compared to pure ILs and organic electrolytes, since they enhance electrolyte stability at high voltages. The electrochemical stability of an IL is a consequence of strong interactions between cations and anions, which effectively hinders electrolyte decomposition at high voltages^22^. In addition, we performed LSV at other IL loadings (Supplymentary (S4)).

We next examined the effects of ILs as co-salts by cyclic voltammetry (CV) at a scan rate of 2 mV s^−1^ to a voltage of 3.7 V. The CV traces reveal which electrolyte-IL concentrations are closest to ideal (Fig. 3(c,d)). EMI-based ion is soft ion that imidazolium aromatic ring which they have occurred charge delocalised, and it affect to improve diffusion of ions. High viscosity leads to reducing mobility of ions, it makes weak interaction between electrode and electrolyte ions in double-layer of device^23^. In this study, the small amount of ILs was added to prevent to affect the ions mobilities and viscosities of the electrolyte. The CV curves demonstrate that ILs have positive effects on electrolytes, which is consistent with the LSV results. Other ILs loadings were also studied (Supplymentary (S5)).

Ion diffusion in the electrolyte from the electrical double layer to the electrode/electrolyte interface is not fast enough during repeated charge/discharge processes, which results in decreases in the specific capacitance of an SC. The transport properties of the electrolyte, such as high ionic conductivity and low viscosity, impact strongly on the operation of the SC. Consequently, these are among the most important properties of an electrolyte^24^. However, the cycling stability of a SC deteriorates due to irreversible ionic reactions that cause the used electrolyte to decompose^25^.

**Figure 4:**
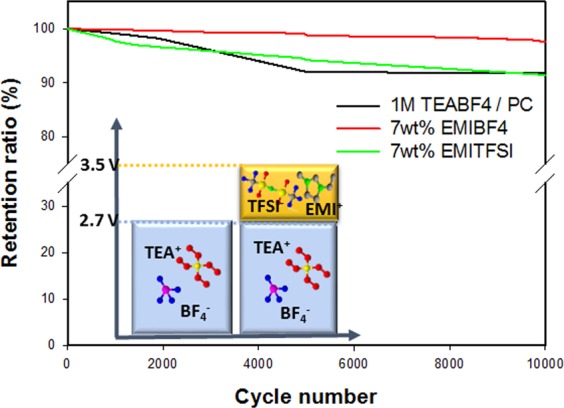
Specific-capacitance retentions of SCs containing electrolytes with ILs as co-salts after 10,000 cycles at 3.5 V.

The improved stabilities of the electrolytes in this study led to increases in SC cycling stabilities at high voltages (Fig. 4). The specific-capacitance-retention data reveal that SCs containing 7 wt% of the IL in the electrolyte exhibit practically useful levels of durability over many galvanostatic charge/discharge cycles at current density of 10 mA cm^−2^. The loss of specific capacitance upon addition of 7 wt% EMIBF4 or EMITFSI to the electrolyte was 2.5% and 8.7%, respectively, after 10,000 cycles at 3.5 V, compared to the specific capacitance of the initial discharge. As mentioned above, the use of ILs as co-salts inhibits electrolyte decomposition at high voltage due to the excellent electrochemical stabilities of the ILs. Other ILs loadings were also studied on the same conditions (Supplymentary (S6)). An EMITFSI- or EMIBF4-co-salt-containing SC is potentially a practical energy-storage system capable of stable charging and discharging at high voltages. Charge/discharge plots were measured and illustrated in Supplymentary (S7). In the case of the 7% electrolyte as it was considered to be the most effective, the rate capability (Supplymentary (S8)) was also measured and that is also improved like other characteristics.

## Conclusions

In summary, we showed that the addition of IL co-salts to as electrolyte effectively increases the ionic conductivity of the electrolyte, while improving its transport properties. This is the result of additional ions created by the IL, which increases the ion density and act as co-salts in the electrolyte; consequently, the ESPW of the electrolyte is wider than those of conventional electrolytes, which led to stabilisation of the SC at high voltage (3.7 V). In addition, the specific capacitance of the SC was 97.5% of its initial value after 10,000 cycles when 7 wt% EMIBF4 was added to the electrolyte as a co-salt. These electrolytes, which can be used in SCs for stable operation at high voltages, were manufactured easily and inexpensively through the addition of ILs as co-salts. Further studies on the designs of SCs that operate at high voltages and their applications are under investigation.

## Methods

### Material preparation

The ILs employed 1-ethyl-3-methylimidazolium tetrafluoroborate (EMIBF_4_, KOEI, Japan) and 1-ethyl-3-methylimidazolium bis(trifluoromethylesulfonyl)imide (EMITFSI, IoLiTec, Germany). Electrolytes were prepared by blending these ILs with an organic electrolyte (1 M TEABF_4_/PC) at ILs concentrations that ranged from 4 to 75 wt%. The porous graphene was placed in a vacuum oven overnight at 120 °C, in order to remove residual moisture, prior to use.

### Characterization

The ion conductivity was measured by using conductivity USB isoPod (eDAQ, Australia) and conductivity probe. Before use, measure the conductance of a solution of kwon value (0.1 mol L-1 KCl).

### Electrochemical measurements

The rubber electrodes consisted of 90 wt% prepared porous graphene as an active material and 10 wt% polytetrafluoroethylene (D60, Daikin Industries, Japan) as a binder. The prepared electrodes were perforated, creating 12-mm in diameter holes, and subsequently dried in a vacuum oven at 120 °C overnight. The working electrode and counter electrode weighed about 11.5 mg each. A CR2032 stainless steel coin cell was used to measure the electrochemical characteristics of the different electrolytes: electrolytes with ILs as additives, organic electrolytes, and pure ILs. The coin cell, which had 2 symmetrical electrodes, was assembled in a glovebox filled with Ar gas.

A 3-electrode system was used for the ESPW measurements. The working electrode was glassy carbon, the counter electrode was Pt metal, and the reference electrode was Ag/Ag+ . For comparison, SCs and 3-electrode systems with the same size and electrodes, but organic electrolyte and pure ILs instead were also test. The compositions of the cell and 3-electrode system used in this study are illustrated in Fig. S2. Cyclic voltammetry (CV) measurements were performed using a potentiostat (EC-Lab, France) at a voltage range of 2.7–3.7 V. Linear sweep voltammetry (LSV) measurements were carried out using the same instrument at a voltage range from −4 V to 4 V and a scan rate of 2 mV s-1. Galvanostatic charge/discharge analysis was performed using a battery tester (Maccor, Series 4000, USA). The cycling stability was measured in the range of 0–3.5 V and under a current density of 5 mA cm^−2^ for 10,000 cycles. All measurements on the electrochemical characteristics were carried out at room temperature and more details illustrated in Supplymentary (S2).

## Supplementary information

Supplymentary

## References

[1] Burke A (2007). R&D considerations for the performance and application of electrochemical capacitors. Electrochim. Acta.

[2] Endres, F., Abbott, A. & MacFarlane, D. R. *Electrodeposition from ionic liquids*, John Wiley & Sons, 2017.

[3] Olson EJ, Bühlmann P (2013). Unbiased Assessment of Electrochemical Windows: Minimizing Mass Transfer Effects on the Evaluation of Anodic and Cathodic Limits. J. Electrochem. Soc..

[4] Armand M, Endres F, MacFarlane DR, Ohno H, Scrosati B (2009). Ionic-liquid materials for the electrochemical challenges of the future. Nat. Mater..

[5] Balducci A (2005). Cycling stability of a hybrid activated carbon//poly(3-methylthiophene) supercapacitor with *N*-butyl-*N*-methylpyrrolidinium bis(trifluoromethanesulfonyl)imide ionic liquid as electrolyte. Electrochim. Acta.

[6] Zhang L, Tsay K, Bock C, Zhang J (2016). Ionic liquids as electrolytes for non-aqueous solutions electrochemical supercapacitors in a temperature range of 20 °C–80 °C. J. Power Sources.

[7] Balducci A (2007). High temperature carbon–carbon supercapacitor using ionic liquid as electrolyte. J. Power Sources.

[8] Balducci A, Soavi F, Mastragostino M (2006). The use of ionic liquids as solvent-free green electrolytes for hybrid supercapacitors. Appl. Phys. A.

[9] Jänes A, Eskusson J, Thomberg T, Romann T, Lust E (2016). Ionic liquid-1,2-dimethoxyethane mixture as electrolyte for high power density supercapacitors. J. Energy Chem..

[10] Ruiz V, Huynh T, Sivakkumar S, Pandolfo A (2012). Ionic liquid–solvent mixtures as supercapacitor electrolytes for extreme temperature operation. RSC Adv..

[11] Guerfi A (2010). Improved electrolytes for Li-ion batteries: Mixtures of ionic liquid and organic electrolyte with enhanced safety and electrochemical performance. J. Power Sources.

[12] Shi M, Kou S, Yan X (2014). Engineering the Electrochemical Capacitive Properties of Graphene Sheets in Ionic‐Liquid Electrolytes by Correct Selection of Anions. ChemSusChem.

[13] Largeot C (2008). Relation between the Ion Size and Pore Size for an Electric Double-Layer Capacitor. J. Am. Chem. Soc..

[14] Xia J, Chen F, Li J, Tao N (2009). Measurement of the quantum capacitance of graphene. Nat. Nanotechnol.

[15] Zheng J, Jow T (1997). The Effect of Salt Concentration in Electrolytes on the Maximum Energy Storage for Double Layer Capacitors. J. Electrochem. Soc..

[16] Park S, Kim K (2017). Tetramethylammonium tetrafluoroborate: The smallest quaternary ammonium tetrafluoroborate salt for use in electrochemical double layer capacitors. J. Power Sources.

[17] Galiński M, Lewandowski A, Stępniak I (2006). Ionic liquids as electrolytes. Electrochim. Acta.

[18] Zhao H, Liang Z-C, Li F (2009). An improved model for the conductivity of room-temperature ionic liquids based on hole theory. J. Mol. Liq..

[19] Xu B (2008). Highly mesoporous and high surface area carbon: A high capacitance electrode material for EDLCs with various electrolytes. Electrochem. Commun..

[20] Chen Y, Zhang X, Zhang D, Yu P, Ma Y (2011). High performance supercapacitors based on reduced graphene oxide in aqueous and ionic liquid electrolytes. Carbon.

[21] Hayyan M, Mjalli FS, Hashim MA, AlNashef IM, Mei TX (2013). Investigating the electrochemical windows of ionic liquids. J. Ind. Eng. Chem..

[22] Bonhote P, Dias A-P, Papageorgiou N, Kalyanasundaram K, Grätzel M (1996). Hydrophobic, Highly Conductive Ambient-Temperature Molten Salts. Inorg. Chem..

[23] Lazzari M, Mastragostino M, Soavi F (2007). Capacitance response of carbons in solvent-free ionic liquid electrolytes. Electrochem. Commun..

[24] Barthel J, Neueder R, Roch H (2000). Density, Relative Permittivity, and Viscosity of Propylene Carbonate + Dimethoxyethane Mixtures from 25 °C to 125 °C. J. Chem. Eng. Data.

[25] Pandey G, Hashmi S (2013). Performance of solid-state supercapacitors with ionic liquid 1-ethyl-3-methylimidazolium tris(pentafluoroethyl) trifluorophosphate based gel polymer electrolyte and modified MWCNT electrodes. Electrochim. Acta.

