# Supplementary material for: The effect of ILs as co-salts in electrolytes for high voltage supercapacitors
Source: Sci Rep. 2019 Feb 4;9:1180. doi: 10.1038/s41598-018-37322-y (PMC6361913; doi:10.1038/s41598-018-37322-y)
Supplement: Supplementary file 1 — Supplymentary [file 41598_2018_37322_MOESM1_ESM.docx]

Supporting information

**The effect of ILs as co-salts in electrolytes for high voltage supercapacitors**

Ha-Na Kwon^a,^ ^b^, Su-Jin Jang^a,^ ^b^, Yun Chan Kang^b^, Kwang Chul Roh*^a^

a. Energy Efficient Materials Team, Energy & Environmental Division, Korea Institute of Ceramic Engineering and Technology (KICET), Soho-Ro 101, Jinju-Si, Gyeongnam, 52851, Korea

b. Department of Materials Science & Engineering, Korea University, Anam-dong, Seongbuk-Gu, Seoul, 02841, Republic of Korea.

S1. Properties of the porous graphene used in this study

Porous graphene was purchased from Nanotek (USA). Before use, it was kept in a vacuum oven at 120 °C overnight in order for any remaining moisture to be removed. The specific surface area (SSA) of graphene and the pore size were determined with a gas analyser (Belsorp-Mini II, Japan) and studied further using the N_2_ adsorption–desorption isotherms and non-localized density functional theory (NLDFT). The SSA of porous graphene was found to be 3481 m^2^/g and the pore size was ranging from 0.7 to 4 nm (Fig. S1). Considering the distribution of these pores, the ILs with a larger ion size compared to organic electrolytes more easily permeate the electrode. Therefore, porous graphene is suitable for SCs, which are prepared with the use of ILs as co-salts.

Figure. S1 NLDFT of porous graphene

S2. Supercapacitor (SC) cell fabrication procedure and measurement method


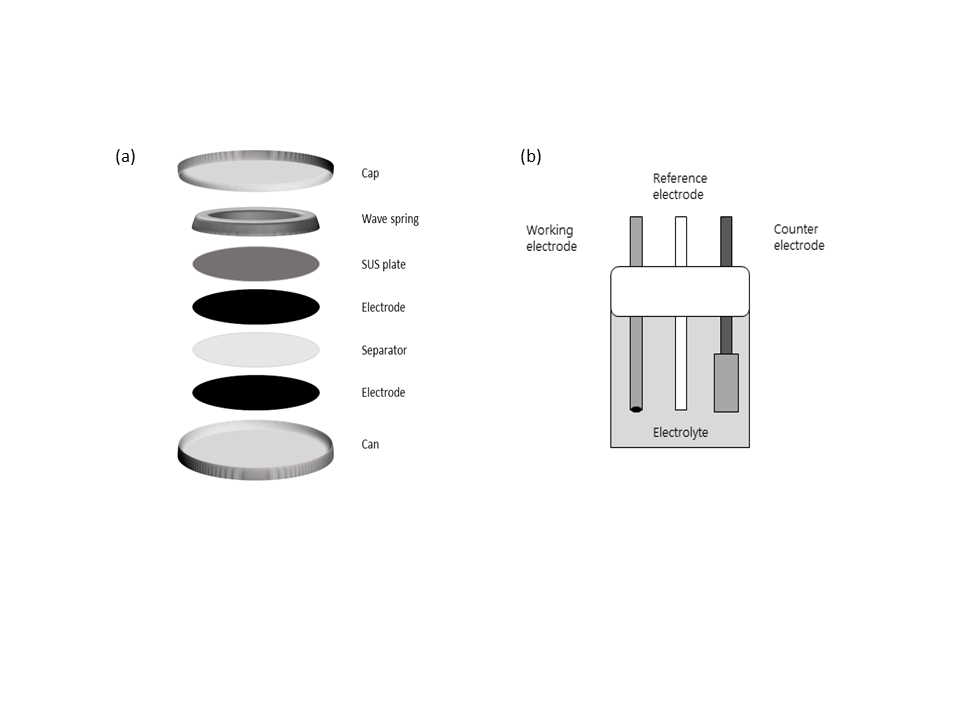


Figure. S2 Compositions of the (a) 2032-coin cell and (b) 3-electrode system.

S3. Relationship between the ion conductivity and viscosity

The molar conductivity (Λ) is associated with the diffusion coefficient (D) of ions in a classical electrolyte, as shown in the Nernst-Einstein equation^1^:

$\Lambda=\frac{z^{2}e_{0}FD}{k_{B}T}=\frac{z^{2}N_{A}e_{0}^{2}D}{k_{B}T}$ [1]

where z is the number of charge carriers, e_0_ is the elementary charge, N_A_ is Avogadro’s number, k_B_ is the Boltzmann constant, and F is the Faraday constant. These factors dependent on the electrolyte since each value is inherent to the electrolytes.

The diffusion coefficient was obtained using the Stokes-Einstein equation^2^:

$D=\frac{k_{B}T}{\sigma\pi r\eta}$ [2]

where T is the temperature, η is the viscosity, and r is the ionic radius of electrolyte.

Equation 3 shows that the molar conductivity is related to the electrical conductivity^3^:

$\sigma=\frac{\Lambda\rho}{M_{w}}$ [3]

where ρ is the density and M_W_ is the molecular weight of the electrolyte.

The electric conductivity could be expressed in the following way by employing the aforementioned three equations:

$\sigma=\frac{z^{2}N_{A}e_{0}^{2}\rho}{\sigma\pi r\eta M_{w}}$ [4]

From this equation, it could be determined that the electric conductivity is inversely proportional to the viscosity, molecular weight, and size of the electrolyte.

S4. Linear sweep voltammetry and cyclic voltammetry of the electrolytes


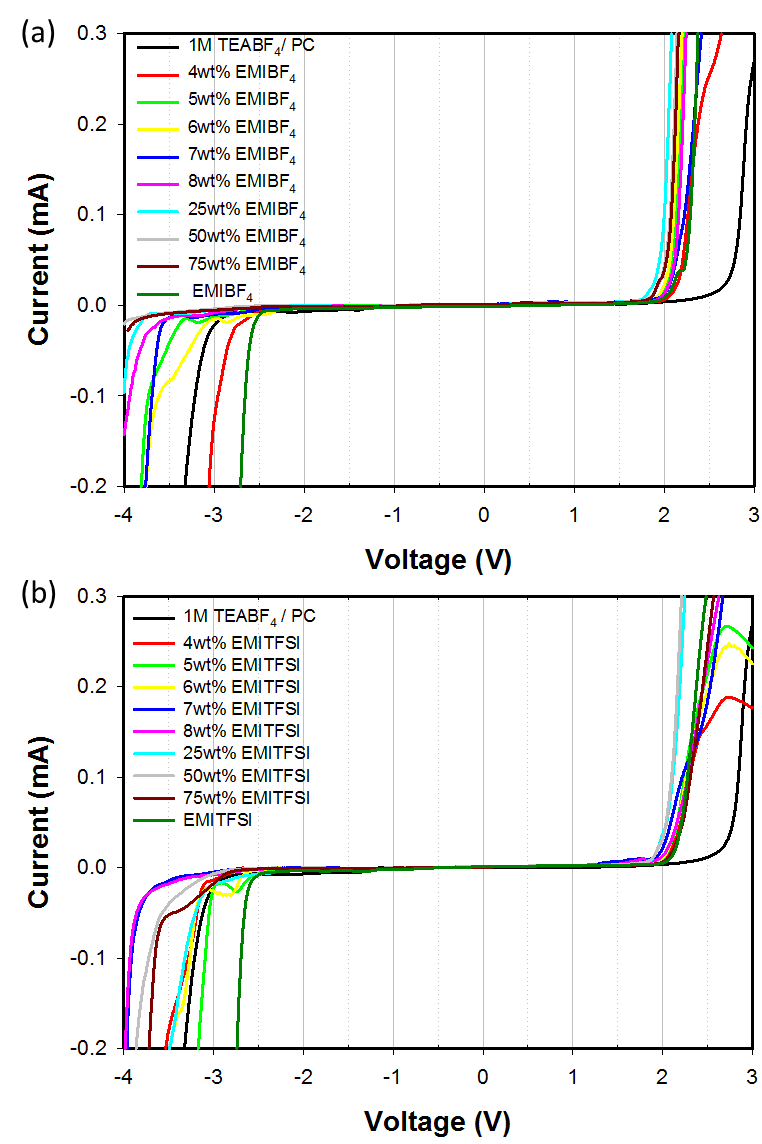


Figure. S3 linear sweep voltammetry of electrolytes used ILs as co-salt (a) EMIBF_4_ (b) EMITFSI

S5. CV curves of SC for the different weight ratios of ILs in the electrolytes


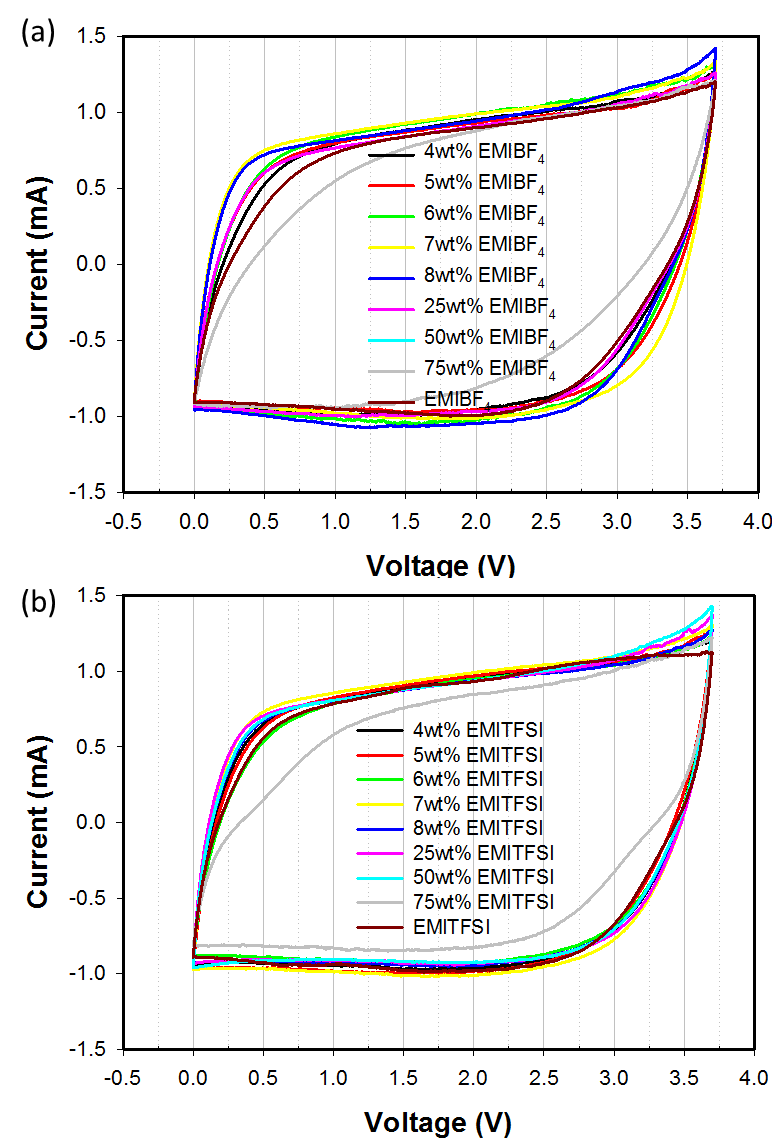


Figure. S4 Cyclic voltammetry of electrolytes used ILs as co-salt at 3.7 V (a) EMIBF_4_ (b) EMITFSI

S6. Cycling stability

| Electrolyte | Retention ratio (%) | |
| --- | --- | --- |
|  | after 5,000 cycles | after 10,000 cycles |
| 1 M TEABF4 / PC | 92.1 | 91.3 |
| 4 wt.% EMIBF_4_ | 92.7 | 85.2 |
| 7 wt% EMIBF_4_ | 98.9 | 97.5 |
| 25 wt% EMIBFI | 93.2 | 93.0 |
| 4 wt% EMITFSI | 98.6 | 90.8 |
| 7 wt% EMITFSI | 94.3 | 91.8 |
| 25 wt% EMITFSI | 91.1 | 78.9 |

Table 1 Retention ratios of the supercapacitors containing an electrolyte with ILs as co-salts after 5,000 and 10,000 cycles at 3.5 V.

S7. Gavanostatic charge/discharge plots


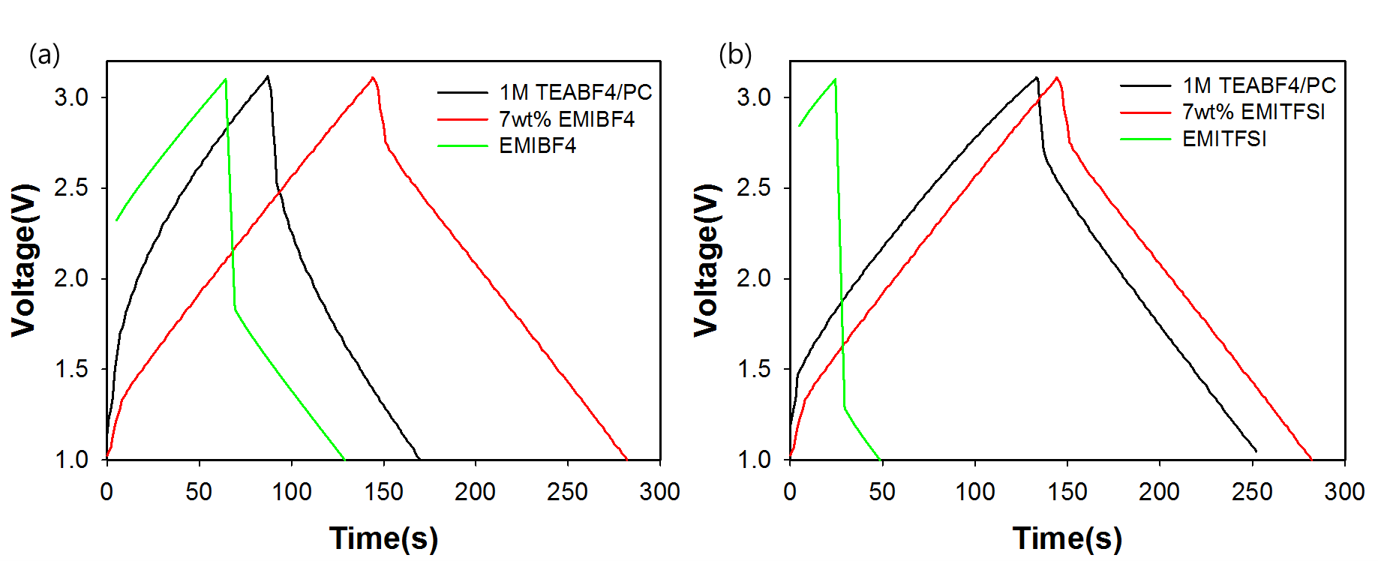


Figure. S5 Galvanostatic charge/discharge plots of electrolytes used ILs as co-salt at 3.5 V (a) EMIBF_4_ (b) EMITFSI

S8. Rate capability of SC for the different weight ratios of ILs in the electrolytes


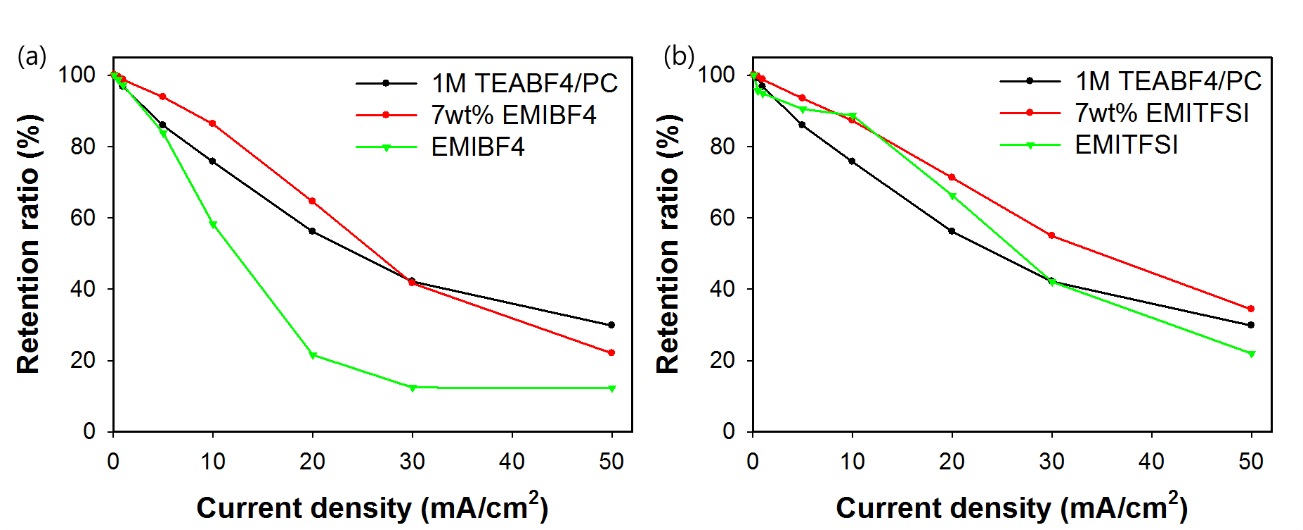


Figure. S6 Rate performance of electrolytes used ILs as co-salt at 3.5 V (a) EMIBF_4_ (b) EMITFSI

References

[1] M. Galiński, A. Lewandowski, and I. Stępniak, *Electrochim. Acta*, **51**, 5567 (2006)

[2] P. Hapiot and C. Lagrost, *Chem. Rev.*, **108**, 2238 (2008)

[3] H. Zhao, Z.-C. Liang, F. Li, *J. Mol. Liq*., **149**, 55 (2009)
